# What Can Be Learnt about Disease Progression in Breast Cancer Dormancy from Relapse Data?

**DOI:** 10.1371/journal.pone.0062320

**Published:** 2013-05-06

**Authors:** Lisa Willis, Trevor A. Graham, Tomás Alarcón, Malcolm R. Alison, Ian P. M. Tomlinson, Karen M. Page

**Affiliations:** 1 Centre for Mathematics and Physics in the Life Sciences and EXperimental Biology, University College London, London, United Kingdom; 2 Sainsbury Laboratory, University of Cambridge, Cambridge, United Kingdom; 3 Center for Evolution and Cancer, Helen Diller Family Comprehensive Cancer Center, Department of Surgery, University of California San Francisco, San Francisco, California, United States of America; 4 Centre de Recerca Matemática, Campus de Bellaterra, Barcelona, Spain; 5 Centre for Tumour Biology, Barts Cancer Institute, Barts and The London School of Medicine and Dentistry, London, United Kingdom; 6 Molecular and Population Genetics, Wellcome Trust Centre for Human Genetics, University of Oxford, Oxford, United Kingdom; 7 Department of Mathematics, University College London, London, United Kingdom; Health Canada, Canada

## Abstract

Breast cancer patients have an anomalously high rate of relapse many years–up to 25 years–after apparently curative surgery removed the primary tumour. Disease progression during the intervening years between resection and relapse is poorly understood. There is evidence that the disease persists as dangerous, tiny metastases that remain at a growth restricted, clinically undetectable size until a transforming event restarts growth. This is the starting point for our study, where patients who have metastases that are all tiny and growth-restricted are said to have cancer dormancy. Can long-term follow-up relapse data from breast cancer patients be used to extract knowledge about the progression of the undetected disease? Here, we evaluate whether this is the case by introducing and analysing four simple mathematical models of cancer dormancy. These models extend the common assumption that a random transforming event, such as a mutation, can restart growth of a tiny, growth-restricted metastasis; thereafter, cancer dormancy progresses to detectable metastasis. We find that physiopathological details, such as the number of random transforming events that metastases must undergo to escape from growth restriction, cannot be extracted from relapse data. This result is unsurprising. However, the same analysis suggested a natural question that does have a surprising answer: why are interesting trends in long-term relapse data not more commonly observed? Further, our models indicate that (a) therapies which induce growth restriction among metastases but do not prevent increases in metastases' tumourigenicity may introduce a time post-surgery when more patients are prone to relapse; and (b), if a number of facts about disease progression are first established, how relapse data might be used to estimate clinically relevant variables, such as the likely numbers of undetected growth-restricted metastases. This work is a necessary, early step in building a quantitative mechanistic understanding of cancer dormancy.

## Introduction

In breast cancer it is relatively common when compared with other cancers for patients to relapse from metastases appearing at distant sites after exceptionally long periods of remission, up to 25 years [1]. The dynamics of disease progression during the intervening years between resection and relapse is largely unknown [2]. Evidence that the periods of remission are inexplicable by continual growth of metastases [3]–[7] implies that there is some period during which all metastases are not growing–they are growth-restricted at sizes and locations that cannot be detected by non-invasive clinical methods. In this study, such patients are said to have breast cancer dormancy.

Growth restriction of tiny metastases could be due to their inability to recruit extra blood vessels required for further growth (cells are pre-angiogenic) [8]–[14], or to immune surveillance [15], [16], or to cell-cycle arrest of disseminated cells [17], perhaps because the new microenvironment lacks the cues to reverse the epithelial-mesenchymal transition [18], [19]. Disseminated cancer cells are thought to have been found in the blood of breast cancer patients who show no other signs of relapse up to 22 years post-resection [20], and these cells are thought to have a short life-span [20], which would indicate that long-term dormancy in breast cancer is maintained by micrometastases that contain proliferating cells rather than by solitary quiescent cells.

Given the experimental constraints that prohibit the study of dormancy *in vivo*, a few recent efforts have attempted to infer the physiopathological mechanisms underlying dormancy from trends in relapse statistics collected from large, long-term follow-up cohorts of patients with previously resected cancers [21], [22]. The approach is analogous to that initiated by Armitage and Doll in the 1960 s, who attempted to infer the number of rate-limiting events that occurred during carcinogenesis from age-onset data [23]. Following Armitage and Doll, more recent work proved that definitive estimates of this number are confounded by clonal expansions that occur as part of the carcinogenic process (see [24] and references therein) and as such, the physiopathological information extractable from the age-incidence data is limited. It seems pertinent to ask what information about the progression of long-term metastatic disease can be confidently inferred from relapse statistics.

Here, we address this question in the context of breast cancer dormancy by introducing and analysing four simple probabilistic models, each motivated by the clinical literature. These models have in common one key assumption, that all patients who relapse beyond a specified minimum time 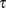 years post-resection have metastases that each underwent at least one period of growth restriction as a micrometastasis until a random growth event, such as a (epi)mutation among the proliferating cells, caused escape from growth restriction. Otherwise the different models represent different combinations of the following physiopathological events: (1) Micrometastases may disappear–the spontaneous disappearance of small cancers and metastases is known to happen (e.g. [25]–[27]); it is often attributed to immune attack or random fluctuations in the balance of cell proliferation and death. (2) Micrometastases may disseminate cells that seed secondary micrometastases at distant sites–this is plausible because the cell-initiators of micrometastases are likely to have high metastatic potential, and breast cancer dormancy patients commonly have circulating tumour cells in their blood [20]. (3) Micrometastases undergo two rate-limiting, random growth events, rather than just one–there is evidence for two significant periods of growth restriction, as solitary cells [17] and as pre-angiogenic micrometastases [8], that may follow in succession in breast cancer dormancy [28]. (4) The rate of escape from growth restriction may change over time–for example, if over generations selection operates on the cells of the micrometastases to increase the cell proliferation rate.

Each model makes simplifying assumptions: the physiopathological events (1)–(4) are each represented by parameters that coarse-grain many, largely unknown, distinct cellular events; each model ignores fluctuations in cell number and intra-heterogeneity within micrometastases; each model ignores the inter-heterogeniety between micrometastases and their respective locations; after a random growth event, micrometastases grow to a clinically detectable size over a fixed growth time 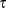 that is common to all micrometastases; micrometastases are assumed not to influence one another's progression. Lastly, the effect of treatments on disease progression is not explicitly modelled, although it may be possible to surmise how treatments will affect relapse rates from their likely effect on the parameters representing (1) – (4) (see, for example, the third section of the results). We return briefly to these assumptions in the discussion.

Despite the simplifications, the models can account for the different trends in relapse data. Their simplicity renders them analytically tractable, and this permitted a full characterisation of how the disease's hidden dynamics translates into trends in relapse data, and so led to a few surprising conclusions that are summarised in the discussion. The characterisation would have been considerably more difficult and perhaps impossible to achieve with more complex models.

## Materials and Methods

### Models

**Figure 1:**
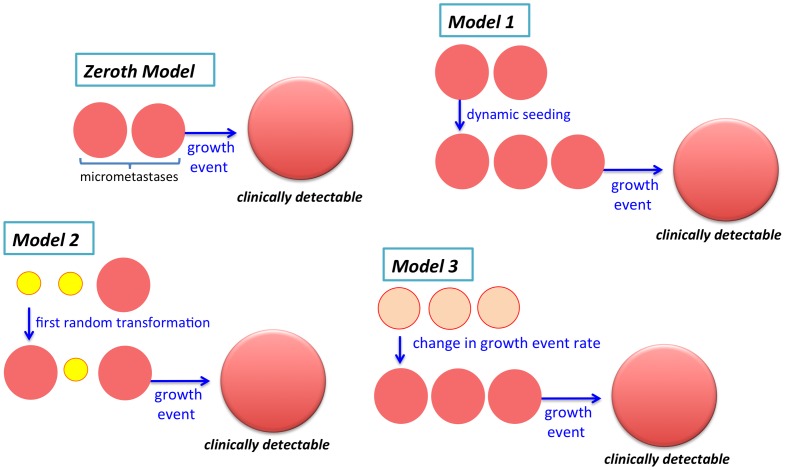
Models. The four models describe four different scenarios for how a patient, who at the time of resection has no detectable or growing cancers, progresses between the states of dormancy, clearance, growth, and relapse.

Patients post-resection can be in one of four states: all metastases in growth restriction as micrometastases (*dormancy*); one or more growing metastases (*growth*); detectable metastases (*relapse*); or no residual cancer (*clearance*). The following models, illustrated in Figure 1, describe four different scenarios for how a patient, who at the time of resection has no detectable or growing cancers, progresses between these states. Their specifications as continuous-time Markov processes are in File S1. In each model, the total number of micrometastases in a patient at time 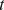 post-resection is 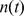 (or 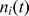, 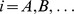 if there are different types 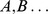 of micrometastases). Notation is summarised in Table 1.

**Table 1:** Abbreviations and notation.

RFI	(breast cancer) recurrence-free interval
EBCTCG	Early Breast Cancer Trials Collaborative Group
ER +/−	estrogen receptor positive/negative
*f_τ_*[*t*+*τ*]	RFI curve at time *t*+*τ* post-resection, normalized at time *τ*
*h*[*t*+*τ*]	Hazard rate at time *t*+*τ* post-resection
E*_t_*[·], Var*_t_*[·]	Average, variance at time *t* post-resection among patients without growing or detectable metastases
*n*(*n_i_*, i = *A*, *B*,…)	Number of micrometastases (of type *i* = *A*, *B*,…)

#### Zeroth Model : micrometastases escape from growth restriction in one rate-limiting step

Upon resection the patient has 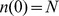 growth-restricted micrometastases. If 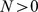, the patient has dormancy; otherwise 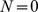 and the patient is cleared of cancer. Each micrometastasis undergoes a random growth event at steady average rate 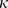 per year, so the total rate of escape from growth restriction at time 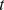 is 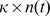; in the Zeroth Model 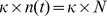, i.e. the risk to the patient remains constant until a growth event. If the growth event is a mutation, 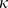 coarse-grains the likelihood of the mutation per cell proliferation, the cell proliferation rate, and the number of proliferating cells in a micrometastasis. Following this event, it is only a matter of time, denoted by a specified growth time 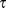, until the micrometastasis grows to a clinically detectable size and the patient relapses. The Zeroth Model has 2 parameters 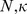.

Models 1 to 3 generalize the Zeroth Model in three different ways.

#### Model 1 : the cells of micrometastases can seed secondary micrometastases and micrometastases can disappear

In Model 1 [22], the *N* micrometastases present upon resection are subject not only to growth events occurring at rate 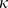 per year, in addition they may disappear at rate 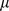 per year, or their disseminated cells may seed new, growth-restricted, secondary micrometastases at rate 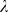 per year. In this way the number of micrometastases 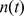 may change with time 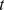: the risk to the patient increases as new micrometastases are seeded or decreases as micrometastases disappear. The parameter 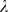 coarse-grains many cellular events, including cell dissemination and colonization in a new environment. The metastatic potential of breast cancer cells depends on the micro-environment from which they originate [29]; we assume that only some micro-environments permit seeding of secondary micrometastases. Another parameter 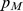 accounts for the frequency with which micrometastases are independently seeded in micro-environments that permit secondary metastasis; the number of micrometastases in such environments is denoted 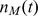.

Model 1 has 5 parameters 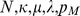.

#### Model 2 : micrometastases must undergo two rate-limiting steps to escape from growth restriction

In Model 2, micrometastases are of two types. Type one, of number 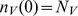 upon resection, must undergo one random growth event to grow to a detectable size; the growth event occurs at steady rate 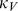 per micrometastasis per year. Type two, of number 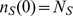 upon resection, must undergo *two* random growth events to grow to a detectable size. The first growth event occurs at rate 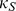 per micrometastasis per year, the second growth event occurs at rate 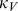 per micrometastasis per year; when such micrometastases undergo a first growth event, the patient still has dormancy, but the risk to the patient increases. Model 2 is motivated by the following scenario [21]: growth-restricted quiescent cell clumps (micrometastases in state 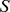) suddenly proliferate to grow to pre-angiogenic micrometastases (micrometastases in state 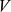); micrometastases seeded by the primary tumour can be in either state 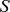 or state 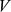. Micrometastases cannot disappear, nor is there dynamic seeding of secondary micrometastases.

Model 2 has 4 parameters 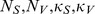.

#### Model 3 : micrometastases escape from growth restriction at a rate that changes with time

In Model 3, the rate of escape from growth restriction is no longer steady: it varies with time in each micrometastasis as 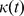 per year because, for example, there is a gradual selection among the cells of each micrometastasis which gradually increases the cell proliferation rate and so the growth event rate. For data fitting, it is simplest to assume that 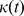 is linear 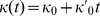. Here, all micrometastases, of number 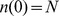 upon resection, are of the same type, and again micrometastases cannot disappear nor is there dynamic seeding of secondary micrometastases.

Model 3 has 3 parameters 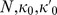.

### Analysis

Relapse data are systematically summarized by Kaplan-Meier recurrence-free interval curves (from hereon referred to as RFI curves) which show the post-resection time evolution of the fraction of patients who do not have recurrent breast cancer as it is defined below in the section `Relapse data'. Throughout, 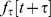 denotes a RFI curve at 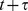 years post-resection normalized at time 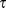 (the normalization time is indicated by the subscript, 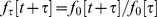). Using RFI curves only after the micrometastases' growth time 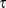 excludes from our analyses patients who already had growing or detectable metastases upon resection. The hazard rate 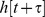 is the rate of patient relapse among those who have survived recurrence-free until that time, and so is related to the RFI curve by 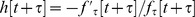.

#### Expressions that relate the models' variables to RFI curves and hazard rates

For each model, the statistics 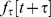 and 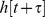 are expressible in terms of the model's variables and parameters. Recall that 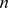 denotes the number of micrometastases. The expressions below are derived in File S1.
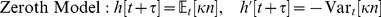
(1)

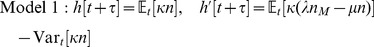
(2)

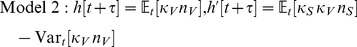
(3)

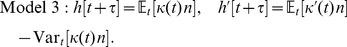
(4)


The expectation 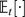 and the variance 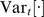 are over patients *without growing or detectable metastases at time 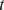 post-resection*. (Note that (1) – (4) relate hazard rates at time 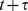 post-resection to 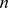 at time 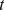 post-resection.) Under the assumption that upon resection parameters are independent, we have.
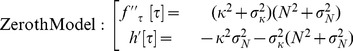
(5)

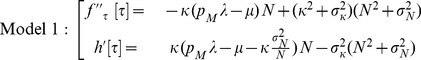
(6)

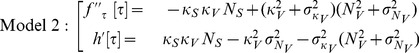
(7)

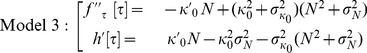
(8)where here 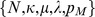, 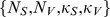, and 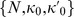 each represent the corresponding population averages among patients, and 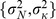, 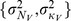, and 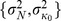 each represent the population variance of the parameter indicated by the subscript (compare (1) – (4)). In data fitting and the presentation of results, it is assumed that 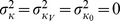 (all patients have the same 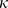 or 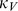), and that the number of micrometastases upon resection is Poisson-distributed with mean 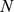 (or with means 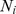, 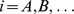, if micrometastases have different types). The latter assumption is equivalent to the very reasonable assumption that the micrometastases extant upon resection were all seeded by a primary tumour which seeds metastases as a time-varying Poisson process. The former assumption is evaluated in the results and in the discussion.

### Relapse Data

Models are fitted to relapse data from the following long-term follow-up studies of relapse among breast cancer patients who did not receive adjuvant therapy : 1) two data sets from the Early Breast Cancer Trialists Collaborative Group (EBCTCG), a 15-year follow-up from 6399 female patients [30]; 2) two data sets from Chia et al., a 10-year follow-up from 1,187 lymph-node negative, lymphovascular negative female patients [31]. The two EBCTCG data sets are women aged less than 50 years at diagnosis and women aged 50–69 years at diagnosis. The two Chia et al. data sets are distinguished by the estrogen receptor positive (ER+) or negative (ER−) status of the primary tumour. This indicates a patient's likely response to endocrine therapies such as tamoxifen. The software GraphClick [32] was used to extract RFI curves from these studies.

The models of dormancy are suitably applied to breast cancer relapse data that specifies patients' recurrence-free intervals (RFIs) [33]. This term was introduced in 2007 [33] to describe patient relapse data where relapses or `recurrences' or `end points' are defined to be any one of the following: local/regional invasive recurrence; distant recurrence; invasive ipsilateral breast tumour recurrence (these are presumed to be a recurrence); death from breast cancer before a recorded relapse. Among studies carried out prior to 2007 (and possibly since 2007), there are often discrepancies in the definitions of breast cancer relapses [33]. In the Chia et al. study, relapses were defined as either the first local (breast or chest wall), regional (ipsilateral axillary, infraclavicular, internal mammary or supraclavicular), or distant recurrence, or death from breast cancer before a recorded relapse, while new contralateral breast cancers were not included. In the EBCTCG study, relapses were defined as the first reappearance of breast cancer at any site, and so new contralateral cancers were included along with the other ‘end points’ specified in [33]. The EBCTCG and Chia et al. data were used because they are from large patient cohorts which ensured that RFI curves were sufficiently smooth for trends in the data to be apparent. The analysis presented in this article leads to methods for establishing results that are quick and easy to repeat on new large cohort data sets as they become available. In the future, as it becomes possible, the models are most suitably applied to large cohort data sets for which it has been unambiguously established that relapses are due to the original tumours. However, applying this stricter definition to relapse data should not alter the results or conclusions in this article.

### Model Fitting

For each model and relapse data set, fitting was by a Monte Carlo method: fitted parameter values are the points in parameter space from a random sample of 10^6^ which gave the minimum total squared deviation between model and data. Points were sampled from a uniform distribution over the following volumes: for the Zeroth Model, 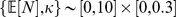; for Model 1, 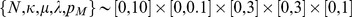; for Model 2, 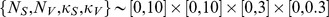; for Model 3, 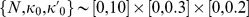. These volumes are sensible, and our conclusions are independent of them. For each randomly sampled point, the total squared deviation between the model's RFI curve (explicit functional forms of RFI curves are derived for each model in File S1) and the data set was recorded. The growth time 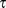 was specified as 3 years for EBCTCG data and 1.5 years for Chia et al. data; these choices for 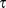 are sensible, see File S1), and for these specific data sets they demonstrate our results optimally, while our conclusions are independent of the choice.

## Results

### Physiopathological Details about Disease Progression cannot be Inferred by Fitting Models to Relapse Data

**Figure 2:**
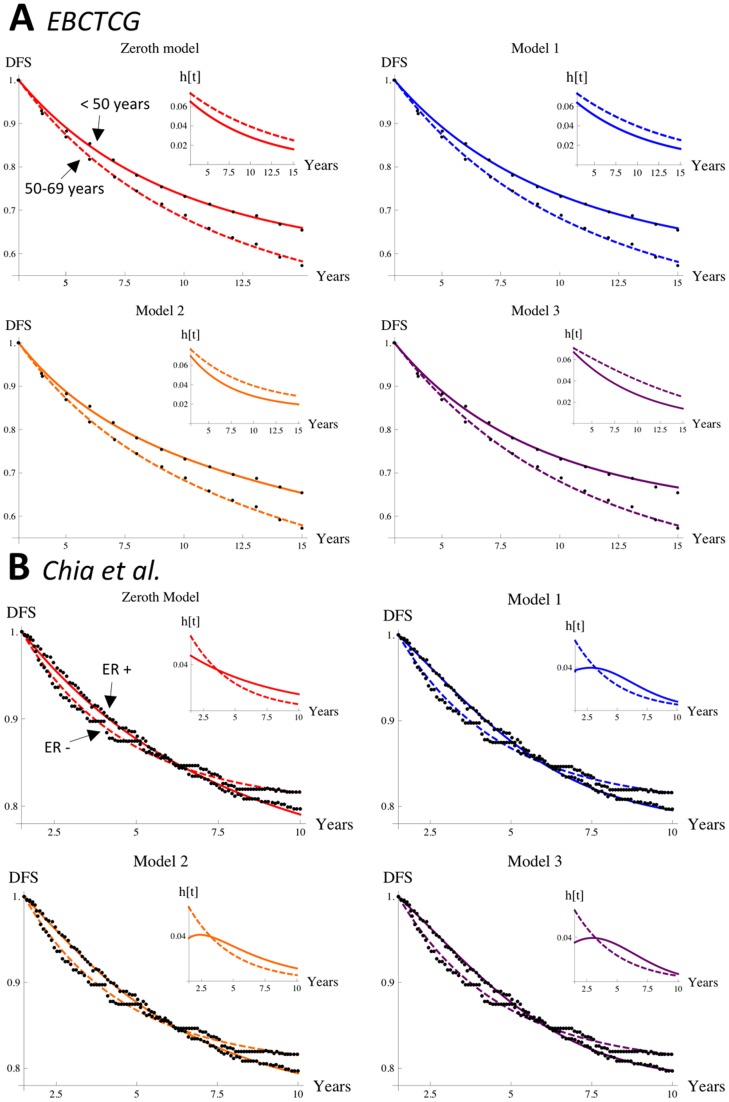
Fitting models to relapse data. The Zeroth Model and Models 1–3 fitted to relapse data from two long-term follow-up studies: patients are grouped by age in the study EBCTCG (Panel A), whereas patients are grouped by ER status in the study Chia et al. (Panel B). Black dots are data points; solids curves are the models for the fitted parameters (Table 2). Hazard rates are inset.

Figure 2 shows the Zeroth Model and Models 1–3 fitted to data from EBCTCG (Panel A) and Chia et al. (Panel B). The graphs in each panel show that each model gives a good fit to both data sets. Hazard rates are inset in each graph; parameters and total squared deviations are in Table 2. There is one notable difference among the fitted models: for the ER+ Chia et al. data set, the hazard rate has a maximum; Models 1–3 can reproduce this maximum but the Zeroth Model cannot, as is explained in the next section. Consequently, Models 1–3 give a better fit than the Zeroth Model (they consistently have a smaller total squared deviation; other discrepancies in the total squared deviations are due to the Monte Carlo fitting method).

**Table 2:** Models fitted to relapse data.

Model	Parameters	Fitted parameters, Total squared deviation	
		**EBCTCG, aged below 50**	**EBCTCG, aged 50–69**
**Zeroth**	{*N*,*κ*}	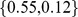 , 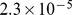	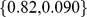 , 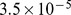
**1**	{*N*, *κ*, *μ*, *λ*, *p_M_*}	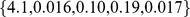 , 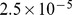	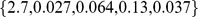 , 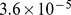
**2**	{*N_s_*, *N_V_*, *κ_s_*, *κ_V_*}	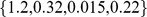 , 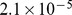	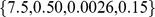 , 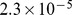
**3**	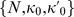	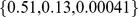 , 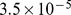	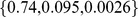 , 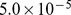
		**Chia et al., ER+**	**Chia et al., ER−**
**Zeroth**	{*N*,*κ*}	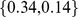 , 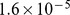	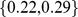 , 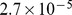
**1**	{*N*, *κ*, *μ*, *λ*, *p_M_*}	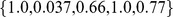 , 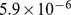	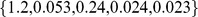 , 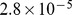
**2**	{*N_s_*, *N_V_*, *κ_s_*, *κ_V_*}	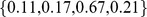 , 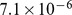	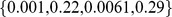 , 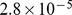
**3**	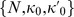	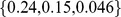 , 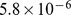	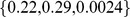 , 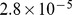

Parameters fitted to EBCTCG relapse data and Chia et al. relapse data for the Zeroth Model and for Models 1–3. The growth time 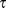 is fixed at 3 years for EBCTCG data and at 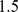 years for Chia et al. data.

### Maxima in Hazard Rates are Expected if the Tumourigenicity of Micrometastases Increases with Time

According to Models 1–3, the hazard rate is increasing at time 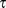 years post-resection whenever the following inequalities are satisfied.
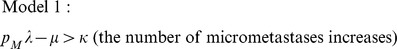


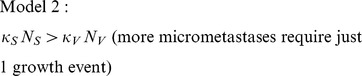


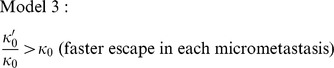
(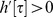 in equations (6) – (8)). The Zeroth Model cannot account for an increase in the hazard rate. The different inequalities get different mechanistic interpretations for increasing hazard rates, but these interpretations have a common base: the risk of escape from growth restriction increases (by the mechanisms in parentheses) before any micrometastasis escapes from growth restriction–we say that the tumourigenicity of micrometastases increases.

Some hazard rates from long-term follow-up breast cancer data are still increasing at times exceeding 5 years post-resection [34]–[36], but a literature survey indicates that presently long-term hazard rates are mostly decreasing or flat (see e.g. Figure 2, and File S1 for a description of how Models 1–3 can account for the extant hazard rates with multiple maxima). Given that cancer cells of micrometastases are thought to be actively dividing, increases in micrometastases' tumourigenicity by some mechanism seems likely. The question then becomes, why are hazard rates from long-term follow-up relapse data sets not more commonly increasing? Expressions (2) – (4) indicate that such trends are obscured by variances among disease course.

### Dormancy-inducing Therapies may Introduce a Period During which More Patients are Prone to Relapse

**Figure 3:**
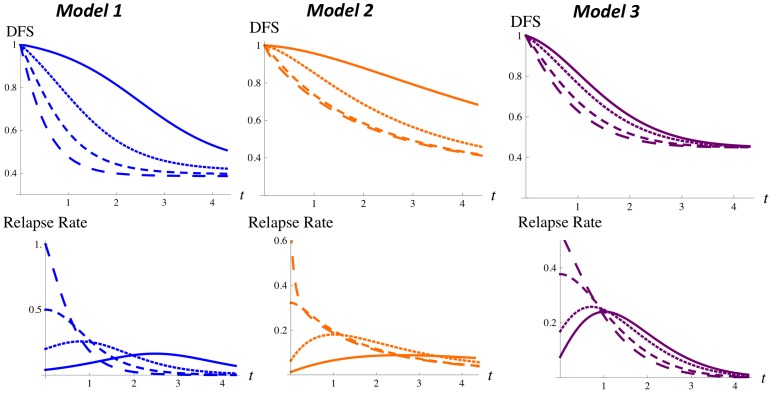
Peaks in the relapse rate. RFI curves (top) and corresponding relapse rates (bottom) for Models 1–3 as parameters 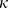 (Model 1)/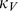 (Model 2)/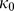 (Model 3) are reduced. In each plot, 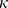/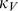/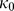 decreases such that the ratio of the right-hand side to the left-hand side of the corresponding inequality determining whether there is a peak in the relapse rate is 2 (long-dashed line), 1 (short-dashed line), 1/5 (dotted line) and 1/25 (solid line).

Models 1–3 produce RFI curves with a maximum in the relapse rate–i.e. a period when more patients are prone to relapse–when the following inequalities are satisfied
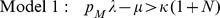


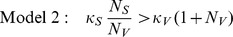


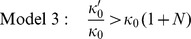
(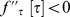 in equations (6) – (8); the relapse rate is 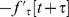), see Figure 3. The Zeroth Model cannot produce RFI curves that have a maximum in the relapse rate. These inequalities are more likely to be satisfied as 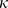 or 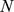 (Model 1), 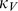 or 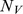 (Model 2), 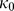 or 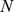 (Model 3) decrease. Therefore, each model indicates that therapies administered upon resection which either (a) induce a period of growth restriction of metastases or (b) eliminate micrometastases that require just one random growth event to escape from growth restriction, but which do not prevent cancer cell proliferation and so prevent increases in micrometastases' tumourgenicity, can introduce a period during which there is an increased rate of patient relapse. This does not imply that such therapies will increase the total number of relapsing patients.

### Estimating Long-term Averages and Standard Deviations in the Number of Patients’ Micrometastases

It may often be valid to make the assumption that from 10 years post-resection the tumourigenicity of micrometastases among patients who have no growing or detectable metastases is no longer changing: e.g. in Model 1, all patients with micrometastases that can seed secondary micrometastases have already relapsed; in Model 2, all micrometastases that must undergo two growth events to escape from growth restriction have already undergone one growth event; in Model 3, micrometastases' cell proliferation rate is no longer increasing. Then in equations (1) – (4) the first term on the right is zero; Models 1–3 all collapse to the Zeroth Model, and for a given 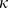 we have



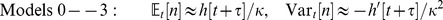
.where in Model 2 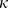 represents 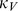. Clearly this approximation can apply only from a time beyond which the hazard rate is non-increasing.

**Figure 4:**
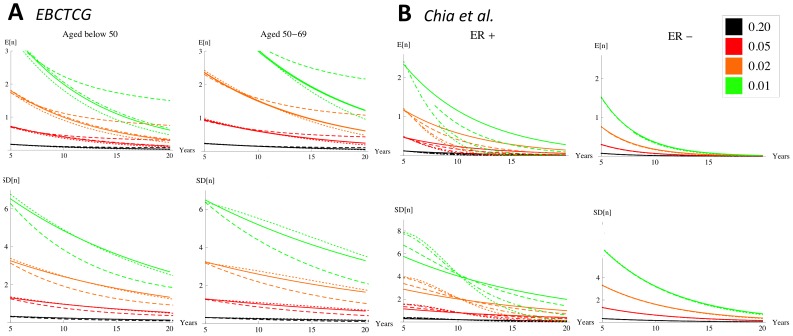
Estimates of means (top row) and standard deviations (bottom row) in patients' numbers of micrometastases. This is up to 20 years post-resection for different values of 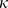: 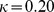 (black), 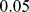 (red), 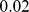 (orange), 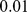 (green). The graphs use extrapolated hazard rates from Models 0–3 (Figure 2) for the EBCTCG data set (panel A) and the Chia et al. data set (panel B). In each plot, graphs that have solid/dot-dashed/long-dash/short-dash lines correspond to Zeroth Model/Model 1/Model 2/Model 3.

The hazard rates from the EBCTCG and Chia et al. data sets (insets, Figure 2) are extrapolated up to 20 years post-resection in order to plot the average 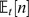 and the standard deviation 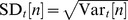 for different values of 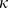, see Figure 4. The plots show that from 10 years post-resection, for both data sets, the averages have a value of one or less and the standard deviations have a value of three or less whenever 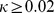 (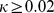 is equivalent to micrometastases escaping from growth restriction within 50 years). This is a quick, approximate method for corroborating our former study [22] which found that long-term breast cancer dormancy can be maintained by small numbers of micrometastases, provided that on average micrometastases escape from growth restriction within a number of years that is less than a human lifetime. Note that if, for a particular data set, a stricter definition of breast cancer recurrence is adopted (as discussed in the methods section `Relapse data'), then the corresponding hazard rate is expected to be reduced. We see from the approximation above that this would strengthen our former conclusion that long-term breast cancer dormancy can be maintained by small numbers of micrometastases: the conditional clause becomes, micrometastases escape from growth restriction within a number of years that can exceed a human lifetime.

## Discussion

Different simple mechanistic models of metastatic progression in cancer dormancy can all account equally well for the different trends in long-term follow-up relapse data. We recommend, therefore, that trends in relapse data be not used as evidence of the physiopathological mechanisms underlying metastatic progression.

If breast cancer dormancy is due to growth-restricted micrometastases that contain proliferating cells, then one might expect the tumourigenicity of the micrometastases, and so the risk to the patient, to increase with time before the micrometastasis escapes from growth restriction, perhaps owing to an evolutionary dynamics among cells of the micrometastasis. If this is the case, then it is intuitive that the hazard rate should increase to a maximum before decreasing and our models corroborate this intuition. Yet hazard rates do not commonly increase–a brief literature survey indicates that, with some exceptions, they are usually flat or decreasing for the entire follow-up period. A more thorough analysis of the models showed that the variability in disease progression among patients tends to produce hazard rates that decrease–and so this variability obscures interesting trends that might otherwise be visible. In the future, as variability decreases because patients are more accurately grouped according to disease status (e.g. HER2 expression), we expect more interesting trends in relapse data to appear. Even then, we would forecast that from such data little can be gleaned about underlying physio-pathological mechanisms. In summary, the models indicate that one explanation for hazard rates which increase for many years post-resection (the increase should continue for a time which is longer than the time it takes for a micrometastasis to grow to a clinically detectable size) is that micrometastases have increasing tumourigenicities; non-increasing hazard rates do not imply that there is no increase in tumourigenicity; no further information can be deduced.

We draw the reader's attention to one observation that is potentially of clinical relevance: our models indicate that therapies administered at around the time of surgery which either (a) induce growth restriction among metastases or (b) reduce the number of growth-restricted metastases, but which do not prevent cell proliferation within metastases (and so prevent increases in their tumourigenicity), may introduce a period during which patients are prone to relapse. Again, this trend–a period during which patients are prone to relapse–will only appear if the variability in disease progression among patients of the cohort is sufficiently low. New adjuvant anti-angiogenic therapies for various cancers [37], [38] that primarily block angiogenic proteins may cause residual cancers to persist at a restricted size of approximately 1 mm in diameter, as has been seen in mouse models of angiogenesis inhibition [8] (see review [37] and references therein). Our study suggests that patients so treated should be monitored at regular intervals for extended times.

This discussion relies on the validity of our key assumption, that cancer dormancy is maintained by growth-restricted micrometastases until a random event, occurring at a time that cannot be precisely predicted, restarts growth to a clinically detectable metastasis. The discussion is based on our analysis of four different models of cancer dormancy. The models are distinguished by different modes of disease progression during dormancy as is now described. In Model 0, there is no disease progression, i.e. the risk to each patient remains constant until a micrometastasis escapes from growth restriction and so the patient no longer has dormancy; in Model 1, disease progression occurs as micrometastases are either newly seeded or eliminated; in Model 2, the chance that micrometastases' escape from growth restriction suddenly and randomly increases owing, for example, to carcinogenic mutations; while in Model 3, the chance that micrometastases' escape from growth restriction changes gradually and deterministically owing, for example, to gradual changes in cell proliferation rates. Each of the models, representing these different modes of disease progression, support our conclusions above.

However, the models were designed to be very simple, and so the inter- and intra-heterogeneity among the micrometastases of a patient, owing to the genotypes or to the locations of micrometastases, were ignored. Further, how relapse rates are affected by variability in the time it takes micrometastases to grow to a detectable size was not investigated, because we opted to concentrate on the dynamics of cancer dormancy which by definition is before any micrometastases escape from growth restriction. Nevertheless, we expect that even when this extra variability is taken into account, then it would not alter the conclusions above. (It is further work to determine how this extra variability alters the estimates of the long-term averages and standard deviations in the number of patients' micrometastases. Inaccuracies in these estimates will also be produced by inaccuracies in the relapse data–see the section `Relapse data').

In order to improve our understanding of breast cancer dormancy, it would be ideal to collect evidence that could be used in conjunction with models like those presented in this article. In the absence of methods for imaging and resecting micrometastases in patients, one possibility is to develop laboratory models, probably mouse models, that permit micrometastases to be observed over a long period and resected and genetically sequenced at different stages of their progression. These laboratory models might be used, for example, to validate our key assumption, to establish whether micrometastases have increasing tumourigenicity, and to improve estimates of the time it takes micrometastases to grow to a detectable size. Genotyping and quantitation of the circulating tumour cells in long-term post-resection breast cancer patients (see e.g. [39]) might also provide a means to determine the risk to a patient from cancer dormancy.

## Supporting Information

File S1 **Supporting information.**
(PDF)
