# Supplementary material for: What Can Be Learnt about Disease Progression in Breast Cancer Dormancy from Relapse Data?
Source: PLoS One. 2013 May 6;8(5):e62320. doi: 10.1371/journal.pone.0062320 (PMC3646031; doi:10.1371/journal.pone.0062320)
Supplement: File S1 — Supporting information. (PDF) [file pone.0062320.s001.pdf]

**What can be learnt about disease progression in breast cancer dormancy from relapse data?**

# Supplementary Material

## Mathematical formulations of the models

### Zeroth Model: micrometastases escape from growth restriction in one rate-limiting step

The patient has a number of micrometastases $n$. Initially, upon resection at time $t = 0$, $n(0) \sim \text{Poisson}(N)$ in all models. In increments of time $\Delta t$, micrometastases undergo growth events as

$$n \to \quad G \quad = \quad \text{growth} \qquad \text{with probability} \qquad \kappa\, n\, \Delta t,$$

probabilities being specified up to $o(\Delta t)$. Here, the number of micrometastases cannot change over time. $n = 0$ corresponds to cancer clearance. For $n \geq 1$, the patient has dormancy until the first growth event. Relapse inevitably follows a growth event once the growth time $\tau$ has subsequently elapsed. In this study, $\tau$ is specified rather than being treated as a free parameter.

Models 1 to 3 generalize the Zeroth Model in three different ways.

### Model 1: the cells of micrometastases can seed secondary micrometastases and micrometastases can disappear

The patient has a number of micrometastases $n$ in total. A subset of these, of number $n_M$, can disseminate cells that seed secondary micrometastases. In increments of time $\Delta t$, the pair $(n, n_M) \in \mathbb{Z}^2_{\geq 0} \cup \{G\}$ changes value as

$$(n, n_M) \to \begin{cases} (n+1, n_M+1) & \text{prob.} \quad p_{_M}\ \lambda\ n_M\ \Delta t, \\ (n+1, n_M) & \text{prob.} \quad (1-p_{_M})\ \lambda\ n_M\ \Delta t, \\ (n-1, n_M-1) & \text{prob.} \quad \mu\ n_M\ \Delta t, \\ (n-1, n_M) & \text{prob.} \quad \mu\ (n-n_M)\ \Delta t, \\ \ G \ = \ \text{growth} & \text{prob.} \quad \kappa\ n\ \Delta t. \end{cases}$$

probabilities being up to $o(\Delta t)$. Relapse inevitably follows a growth event after growth time $\tau$.

### Model 2: micrometastases require two rate-limiting steps to escape from growth restriction

The patient has micrometastases in different states denoted by $S$, $SV$, and $V$ of number $n_S$, $n_{SV}$, and $n_V$ respectively. In increments of time $\Delta t$, the triple $(n_S, n_{SV}, n_V) \in \mathbb{Z}^3{}_{\geq 0} \cup \{G\}$ changes value as

$$(n_S, n_{SV}, n_V) \to \begin{cases} (n_S - 1, n_{SV}+1, n_I) & \text{prob. } \kappa_{_S}\ n_S\ \Delta t, \\ \ G \ = \ \text{growth} & \text{prob. } (\kappa_{_{SV}}\ n_{SV} + \kappa_{_V}\ n_V)\ \Delta t, \end{cases}$$

probabilities being up to $o(\Delta t)$. Relapse inevitably follows a growth event after growth time $\tau$. This definition is more general than that of the main text where $\kappa_{_{SV}} = \kappa_{_V}$

### Model 3: micrometastases escape from growth restriction at a rate that changes with time

In increments of time $\Delta t$, the patient's number of micrometastases $n \in \mathbb{Z}_{\geq 0} \cup \{G\}$ changes value as:

$$n \to \quad G \quad = \quad \text{growth} \qquad \text{prob.} \qquad \kappa(t)\, n\ \Delta t$$

probabilities being up to $o(\Delta t)$.

### The specified growth time $\tau$

For model fitting, the growth time $\tau$ was specified as 3 years for EBCTCG data and 1.5 years for Chia et al. data; the median doubling time in 199 cases of growing primary and metastatic breast cancer was 3.5 months [1], which gives an estimated time of 3 years for micrometastases to grow from 1 mm in diameter to a detectable size; study [2] estimated the metastasis growth time to be only 9 months; in other studies, estimates from the tumour volume-doubling time of breast metastases suggest that $\tau$ is approximately 2.5 years [3]. The choices for $\tau$ are sensible, and for the specific data sets used in this study, they demonstrate the results optimally; the conclusions are independent of the choices for $\tau$. Recall that because relapse data from times $< \tau$ years is excluded from analyses, all patients who already had detectable or growing metastases upon resection are excluded from analyses.

## Supplementary Material for Analyses

### Explicit expressions for RFI curves used for rapid data fitting

In this section, for Models 0–3 we present explicit analytic expressions for RFI curves $f_\tau[t+\tau]$ at time $t+\tau$ post-resection[1], where the subscript denotes normalization at time $\tau$ (necessary in order to exclude from analyses patients with growing or detectable metastases upon resection). These expressions enabled models to be fitted to data rapidly.

The waiting time $T_R$ denotes the time at which a patient relapses, equivalent to the first time at which a metastasis becomes detectable; the waiting time $T_G$ denotes the time at which a growth event first occurs. For a given model of dormancy, parameters of the model may be different in different patients. Then the RFI curve is the probability that patients have not relapsed by time $t+\tau$ averaged among patients, i.e. averaged over parameters of the model according to some prescribed distribution representing parameter values among patients at the time of resection. Expressions are first derived for Models 0, 3, and 2 by elementary probability, then for Model 1 by a more lengthy derivation.

### The Zeroth Model and Model 3

Upon resection a patient has $n(0)$ micrometastases (of a single type) at time $t$, each undergoing growth events at a rate $\kappa(t)$ that may vary with time. This is Model 3, and in the special case $\kappa'(t)=0$ it is the Zeroth Model. By definition,

$$\begin{aligned}\frac{dP\{T_G>t\}}{dt} &= \lim_{\Delta t\to 0}\frac{(P\{T_G>t+\Delta t|T_G>t\}-1)P\{T_G>t\}}{\Delta t}\\ &= \lim_{\Delta t\to 0}\Big(\frac{1-\kappa(t)\,n(0)\,\Delta t - o(\Delta t)-1}{\Delta t}\Big)\,P\{T_G>t\}\\ &= -\kappa(t)\,n(0)\,P\{T_G>t\}. \qquad (1)\end{aligned}$$

By normalizing RFI data at the growth time $\tau$ and analysing it only beyond this time, we are excluding from our analyses all patients who upon resection have either growing or detectable metastases. Initially upon resection at time 0, then, $P\{T_G>0\}=1$, and the unique solution of this differential equation is

$$P\{T_G>t\}=P\{T_R>t+\tau\}=e^{-(\int_0^t \kappa(u)du)\,n(0)}. \qquad (2)$$

[1]A Kaplan-Meier survival curve is derived from a set of times $0\le t_1<t_2<\ldots$ at which time the members of the cohort reach a specified ‘end point’. First, the data is discretized into time intervals $[u_i,u_{i+1})$. If there are $r_i$ surviving patients at time $u_i$ of whom $d_i$ reach the end point in $[u_i,u_{i+1})$, then the Kaplan-Meier curve is

$$\hat{f}[t]=\Pi_{i:u_i<t}\Big(1-\frac{d_i}{r_i}\Big).$$

Now averaging over parameter values upon resection, this gives the explicit expression for the RFI curve normalized at time $\tau$

$$f_\tau[t+\tau] = \mathbb{E}_0[P\{T_R > t+\tau\}] = \mathbb{E}_0[e^{-(\int_0^t \kappa(u)du)\, n(0)}], \quad t > 0. \tag{3}$$

where throughout our analyses and in the main text the expectation operator with a subscript $\mathbb{E}_t[\cdot]$ denotes the average over patients *without growing or detectable metastases at time t post-resection.* In the main text, a linear dependence on time is assumed for $\kappa(t) = \kappa_0 + \kappa_0' t$, then (3) becomes

$$f_\tau[t+\tau] = \mathbb{E}_0[e^{-(\kappa_0 t + \frac{\kappa_0'}{2} t^2)\, n(0)}], \quad t > 0. \tag{4}$$

Assuming also that $\kappa_0$, $\kappa_0'$ take the same values in all patients, and that among patients $n(0)$ is distributed as a Poisson random variable with mean $N$, (4) gives explicit functional forms for the RFI curves of Model 3 and the Zeroth Model.

**Model 2**

In Model 2, the patient's micrometastases must undergo either one growth event to escape from growth restriction (type $V$), or two growth events to escape from growth restriction (type $S$). Here $T_G$ denotes the first time at which a micrometastasis starts growing to a detectable size without any further intervening periods of growth restriction. The number of micrometastases of type $V$ and type $S$ upon resection are denoted by $N_V$ and $N_S$ respectively.

In the case $(n_V(0), n_S(0)) = (1, 0)$, $P\{T_G > t\}$ is again got directly from equation (3), $P\{T_G > t\} = e^{-\kappa_V t}$. When $(n_V(0), n_S(0)) = (0, 1)$, $T_G$ equals $T_S + T_{SV}$, where $T_S$ is the waiting time to the first growth event occurring at steady rate $\kappa_S$ and $T_{SV}$ is the waiting time for the second growth even occurring at steady rate $\kappa_{SV}$. Again, as in (3),

$$P\{T_S > t\} = e^{-\kappa_S t} \qquad \text{and} \qquad P\{T_{SV} > t\} = e^{-\kappa_{SV} t}$$

with corresponding probability density functions (p.d.f.s)

$$p_{T_S}(t) = -\frac{d}{dt} P\{T_S > t\} = \kappa_S e^{-\kappa_S t} \qquad \text{and} \qquad p_{T_{SV}}(t) = -\frac{d}{dt} P\{T_{SV} > t\} = \kappa_{SV} e^{-\kappa_{SV} t}.$$

So in the case $(n_V(0), n_S(0)) = (0, 1)$, the p.d.f. of $T_G$ is $\int_0^t p_{T_S}(u) p_{T_{SV}}(t-u)du = \frac{\kappa_S \kappa_{SV}}{\kappa_S - \kappa_{SV}}(e^{-\kappa_{SV} t} - e^{-\kappa_S t})$, giving

$$P\{T_G > t\} = 1 - \int_0^t p_{T_S}(u) p_{T_{SV}}(t-u)du = \frac{\kappa_S}{\kappa_S - \kappa_{SV}} e^{-\kappa_{SV} t} - \frac{\kappa_{SV}}{\kappa_S - \kappa_{SV}} e^{-\kappa_S t}.$$

From these expressions and the independence of micrometastases, we have

$$f_\tau[t+\tau] = \mathbb{E}_0[P\{T_G > t\}] = \mathbb{E}_0[\prod_{\text{micromet } i} P\{T_{G_i} > t\}] = \mathbb{E}_0\Big[e^{-\kappa_V t\, n_V(0)} \Big(\frac{\kappa_S e^{-\kappa_{SV} t} - \kappa_{SV} e^{-\kappa_S t}}{\kappa_S - \kappa_{SV}}\Big)^{n_S(0)}\Big], \quad t > 0. \tag{5}$$

**Model 1**

The following derivation [4] is a simple extension of work in [5] on a one-type linear birth-death process with killing. It enables rapid simulations of RFI curves for Model 1 by numerically solving Kolmogorov equations using standard software. In the special case $p_M = 1$, there is an explicit analytic expression for RFI curves which can be found in [5].

Let $n_{\not M} = n - n_M$. The stopping times $T_C$ and $T_G$ denote the time of cancer clearance ($n = 0$) and the time of a growth event $n \to G$ respectively. The probability generating function (p.g.f.) of $(n_M, n_{\not M})$ at time $t$ given $(n_M(0), n_{\not M}(0)) = (j,k)$ is denoted by $g_{j,k}[w,z,t] = \mathbb{E}[w^{n_M} z^{n_{\not M}}]$ and the transition probability of $(n_M, n_{\not M})$ such that $(n_M, n_{\not M}) = (l,m) \to (u,v)$ in time $t$ is denoted by $P_{(l,m)\to(u,v)}(t)$. The coefficients of $g_{j,k}[w,z,t]$ tell us everything about the evolution of $(n_M, n_{\not M})$. Importantly,

$$\begin{aligned} P\{T_C < t\} &= P_{(j,k)\to(0,0)}(t) = g_{j,k}[0,0,t]. \\ \text{and} \qquad P\{T_G < t\} &= 1 - \sum_{l\geq 0, m\geq 0} P_{(j,k)\to(l,m)}(t) = 1 - g_{j,k}[1,1,t]. \end{aligned} \tag{6}$$

Assume $\mu > 0$. $(n_M, n_{\not M})$ is eventually either absorbed at 0 (*clearance*) or reaches $G$ (*growth*) (proof not shown). The probability that $(n_M, n_{\not M})$ is eventually absorbed at 0 is

$$\lim_{t\to\infty} P\{T_C < t\} = P\{(n_M, n_{\not M}) \text{ hits 0 before G} \mid \ (n_M(0), n_{\not M}(0)) = (j,k)\} = (s_M)^j (s_{\not M})^k,$$

where

$$\begin{aligned} s_M = \lim_{t\to\infty} P\{\ (n_M(t), n_{\not M}(t)) &= (0,0)\ |(n_M(0), n_{\not M}(0)) = (1,0)\}, \\ s_{\not M} = \lim_{t\to\infty} P\{\ (n_M(t), n_{\not M}(t)) &= (0,0)\ |(n_M(0), n_{\not M}(0)) = (0,1)\} \end{aligned}$$

are the roots $0 \leq s_M, s_{\not M} \leq 1$ of the equations

$$\begin{aligned} x &= \Big(\frac{\mu}{\lambda+\mu+\kappa}\Big) + \Big(\frac{p_M \lambda}{\lambda+\mu+\kappa}\Big)\, x^2 + \Big(\frac{(1-p_M)\lambda}{\lambda+\mu+\kappa}\Big) xy, \\ y &= \frac{\mu}{\mu+\kappa}. \end{aligned} \tag{7}$$

This is because $s_M, s_{\not M}$ must satisfy the following conditioning on the first transition (here $(\cdot,\cdot) \overset{\text{1st}}{\longmapsto} (\cdot,\cdot)$ denotes the first transition and $C|(j,k)$ denotes the event of eventual clearance from state $(n_M, n_{\not M}) = (j,k)$):

$$\begin{aligned} P\{C|(1,0)\} &= P\{C|(0,0)\}P\{(1,0) \overset{\text{1st}}{\longmapsto} (0,0)\} + P\{C|(2,0)\}P\{(1,0) \overset{\text{1st}}{\longmapsto} (2,0)\} \\ &\quad + \ P\{C|(1,1)\}P\{(1,0) \overset{\text{1st}}{\longmapsto} (1,1)\}, \\ &= P\{C|(0,0)\}P\{(1,0) \overset{\text{1st}}{\longmapsto} (0,0)\} + (P\{C|(1,0)\})^2 P\{(1,0) \overset{\text{1st}}{\longmapsto} (2,0)\} \\ &\quad + \ P\{C|(0,1)\}P\{C|(1,0)\}P\{(1,0) \overset{\text{1st}}{\longmapsto} (1,1)\}, \\ P\{C|(0,1)\} &= P\{C|(0,0)\}P\{(0,1) \overset{\text{1st}}{\longmapsto} (0,0)\}. \end{aligned}$$

Above we have used the stationarity and the Markov property of the process which together give $P\{C|\{(j,k) \to (l,m) \text{ in time } t\}\} = P\{C|(l,m)\}$ for all $(l,m)$ and for all time $t \geq 0$. In the second line we have used that $P\{C|(2,0)\} = P\{C|(1,0) \cap C|(1,0)\} = P\{C|(1,0)|C|(1,0)\}P\{C|(1,0)\} = (P\{C|(1,0)\})^2$ as clearance in one instance is independent of clearance in another, and similarly $P\{C|(1,1)\} = P\{C|(1,0)\}P\{C|(0,1)\}$.

The process associated with $(n_M, n_{\not M})$ *conditioned on clearance* is denoted $(\hat{n}_M, \hat{n}_{\not M})$. Let $\hat{P}_{(j,k)\to(l,m)}(t)$ be the corresponding transition probabilities and $\hat{g}_{j,k}[w,z,t]$ be the corresponding p.g.f. The transition probabilities of $(\hat{n}_M, \hat{n}_{\not M})$ are then

$$\begin{aligned} \hat{P}_{(j,k)\to(l,m)}(t) &= P\{\text{transition } (j,k) \mapsto (l,m) \text{ in time } t \mid \ C\} \\ &= \frac{P\{C|(l,m)\}P_{(j,k)\to(l,m)}(t)}{P\{C|(j,k)\}} \\ &= \frac{s_M^l s_{\not M}^m \quad P_{(j,k)\to(l,m)}(t)}{s_M^j s_{\not M}^k}. \end{aligned} \tag{8}$$

From this we see that $(\hat{n}_M, \hat{n}_{\not M})$ transits in infinitesimal time $\Delta t$ as

$$(\hat{n}_M, \hat{n}_{\not M}) \rightarrow \begin{cases} (\hat{n}_M + 1, \hat{n}_{\not M}) & \text{prob.} \quad p_M \lambda s_M \; n_M \; \Delta t \\ (\hat{n}_M - 1, \hat{n}_{\not M}) & \text{prob.} \quad (\mu / s_M) \; n_M \; \Delta t \\ (\hat{n}_M, \hat{n}_{\not M} + 1) & \text{prob.} \quad (1 - p_M) \lambda s_{\not M} \; n_M \; \Delta t \\ (\hat{n}_M, \hat{n}_{\not M} - 1) & \text{prob.} \quad (\mu / s_{\not M}) \; n_{\not M} \; \Delta t, \end{cases}$$

up to $o(\Delta t)$. $(\hat{n}_M, \hat{n}_{\not M})$ is a two-type birth-death process with absorption at $(0,0)$ but no growth, so well known theory applies.

The p.g.f. of $(n_M, n_{\not M})$ in terms of the p.g.f. of $(\hat{n}_M, \hat{n}_{\not M})$ is

$$\begin{aligned} g_{j,k}[w,z,t] &= \sum_{l,m} \hat{P}_{(j,k)\rightarrow(l,m)}(t) s_M^{-l+j} s_{\not M}^{-m+k} w^l z^m, \qquad \text{by equation (8)} \\ &= s_M^j s_{\not M}^k \sum_{l,m} \hat{P}_{(j,k)\rightarrow(l,m)}(t) \Big(\frac{w}{s_M}\Big)^l \Big(\frac{z}{s_{\not M}}\Big)^m \\ &= s_M^j s_{\not M}^k \quad \hat{g}_{j,k}[\frac{w}{s_M}, \frac{z}{s_{\not M}}, t]. \end{aligned}$$

Let $\psi^{(M)}[w,z,t]$ and $\psi^{(\not M)}[w,z,t]$ denote the p.g.f.s of the process $(\hat{n}_M, \hat{n}_{\not M})$ for initial conditions $(1,0)$ and $(0,1)$ respectively, so $\psi^{(M)}[w,z,t] = \hat{g}_{1,0}[w,z,t]$ and $\psi^{(\not M)}[w,z,t] = \hat{g}_{0,1}[w,z,t]$. Since the p.g.f.s of two independent vector-valued random variables $X$ and $Y$ satisfy $g_{X+Y}[z] = \mathbb{E}[z^{X+Y}] = \mathbb{E}[z^X]\,\mathbb{E}[z^Y] = g_X[z]\,g_Y[z]$, then

$$\hat{g}_{j,k}[w,z,t] = \sum_{(l,m)} P_{(j,k)\rightarrow(l,m)}(t)\, w^l\, z^m = (\psi^{(M)}[w,z,t])^j\, (\psi^{(\not M)}[w,z,t])^k, \tag{9}$$

and

$$g_{j,k}[w,z,t] = s_M^j s_{\not M}^k \; \hat{g}_{j,k}[\frac{w}{s_M}, \frac{z}{s_{\not M}}, t] = \Big(s_M \; \psi^{(M)}[\frac{w}{s_M}, \frac{z}{s_{\not M}}, t]\Big)^j \Big(s_{\not M} \; \psi^{(\not M)}[\frac{w}{s_M}, \frac{z}{s_{\not M}}, t]\Big)^k, \tag{10}$$

so it suffices to find convenient expressions for $\psi^{(M)}[w,z,t]$ and $\psi^{(\not M)}[w,z,t]$. We now derive such convenient expressions using a pair of differential equations called the Kolmogorov equations.

The Markov property of $(\hat{n}_M, \hat{n}_{\not M})$ is summarized by the Chapman-Kolmogorov equations

$$\begin{aligned} \hat{P}_{(j,k)\rightarrow(l,m)}(t+t') &= \sum_{r,s=0}^{\infty} \hat{P}_{(j,k)\rightarrow(r,s)}(t) \hat{P}\{\text{transition } (r,s) \mapsto (l,m) \text{ in time } t' \mid (j,k) \rightarrow (r,s) \text{ in time } t\} \\ &= \sum_{r,s=0}^{\infty} \hat{P}_{(j,k)\rightarrow(r,s)}(t) \hat{P}_{(r,s)\rightarrow(l,m)}(t'). \end{aligned}$$

Using this and equation (9), we have

$$\begin{aligned} \psi^{(M)}[w,z,t+t'] &= \sum_{(l,m)} \hat{P}_{(1,0)\rightarrow(l,m)}(t+t') w^l z^m \\ &= \sum_{(l,m)} \sum_{(l',m')} \hat{P}_{(1,0)\rightarrow(l',m')}(t) \hat{P}_{(l',m')\rightarrow(l,m)}(t') w^l z^m \\ &= \sum_{(l',m')} \hat{P}_{(1,0)\rightarrow(l',m')}(t) \sum_{(l,m)} \hat{P}_{(l',m')\rightarrow(l,m)}(t') w^l z^m \\ &= \sum_{(l',m')} \hat{P}_{(1,0)\rightarrow(l',m')}(t) \Big(\psi^{(M)}[w,z,t']\Big)^{l'} \Big(\psi^{(\not M)}[w,z,t']\Big)^{m'} \\ &= \psi^{(M)}\Big[\psi^{(M)}[w,z,t'], \psi^{(\not M)}[w,z,t'], t\Big]. \end{aligned}$$

An analogous derivation for $\psi^{(\not M)}$ shows that the p.g.f.s $\psi^{(M)}$ and $\psi^{(\not M)}$ satisfy

$$\psi^{(M)}[w,z,t+t'] = \psi^{(M)}[\psi^{(M)}[w,z,t'],\psi^{(\not M)}[w,z,t'],t], \tag{11}$$
$$\psi^{(\not M)}[w,z,t+t'] = \psi^{(\not M)}[\psi^{(M)}[w,z,t'],\psi^{(\not M)}[w,z,t'],t]. \tag{12}$$

For convenience of notation now define $\lambda_1 = p_M \lambda s_M, \mu_1 = \mu/s_M, \lambda_2 = (1-p_M)\lambda s_{\not M}$, and $\mu_2 = \mu/s_{\not M}$. By definition

$$\psi^{(M)}[w,z,\Delta t] = \mu_1 \Delta t + (1-(\mu_1+\lambda_1+\lambda_2)\Delta t)w + \lambda_1 \Delta t w^2 + \lambda_2 \Delta t wz, \tag{13}$$
$$\psi^{(\not M)}[w,z,\Delta t] = \mu_2 \Delta t + (1-\mu_2\Delta t)z \tag{14}$$

up to $o(\Delta t)$. Differential equations are got by setting $t' = \Delta t$ in (11) & (12) and substituting the expressions (13) & (14) into the right-hand sides of (11) & (12), then expanding into Taylor series in terms of the increment $\Delta t$; for $\psi^{(M)}$ in (11),

$$\begin{aligned}\psi^{(M)}[w,z,t+\Delta t] &= \psi^{(M)}[\mu_1\Delta t + (1-(\mu_1+\lambda_1+\lambda_2)\Delta t)w + \lambda_1\Delta t w^2 + \lambda_2 \Delta t wz,\ \mu_2\Delta t + (1-\mu_2\Delta t)z,\ t], \\ &= \psi^{(M)}[w,z,t] + \frac{\partial \psi^{(M)}}{\partial w}(\mu_1 - (\mu_1+\lambda_1+\lambda_2)w + \lambda_1 w^2 + \lambda_2 wz)\,\Delta t + \frac{\partial \psi^{(M)}}{\partial z}(\mu_2 - \mu_2 z)\,\Delta t\end{aligned}$$

up to $o(\Delta t)$. Rearranging and letting $\Delta t \downarrow 0$ obtains a PDE. A similar exercise for $\psi^{(\not M)}$ gets a second PDE; together these PDEs are called the Kolmogorov forward equations.

The Kolmogorov backward equations are got by setting $t = \Delta t$ in (11) & (12), then again substituting (13) & (14) into the right-hand sides; for $\psi^{(M)}$ in (11),

$$\begin{aligned}\psi^{(M)}[w,z,\Delta t+t'] &= \psi^{(M)}[\psi^{(M)}[w,z,t'],\psi^{(\not M)}[w,z,t'],\Delta t], \\ &= \mu_1\Delta t + (1-(\mu_1+\lambda_1+\lambda_2)\Delta t)\,\psi^{(M)}[w,z,t'] + \lambda_1\Delta t\,\psi^{(M)}[w,z,t']^2 \\ &\qquad + \lambda_2\Delta t\,\psi^{(M)}[w,z,t']\,\psi^{(\not M)}[w,z,t']\end{aligned}$$

up to $o(\Delta t)$. Rearranging and letting $\Delta t \downarrow 0$ obtains an ODE. A similar exercise for $\psi^{(\not M)}$ gets a second ODE; together these ODEs are called the Kolmogorov backward equations.

In summary, we have

Backward equations:

$$\frac{d\psi^{(M)}}{dt} = \mu_1 - (\mu_1+\lambda_1+\lambda_2)\psi^{(M)} + \lambda_1(\psi^{(M)})^2 + \lambda_2\psi^{(M)}\psi^{(\not M)},$$
$$\frac{d\psi^{(\not M)}}{dt} = \mu_2 - \mu_2\psi^{(\not M)}.$$

Forward equations:

$$\frac{\partial\psi^{(M)}}{\partial t} = \frac{\partial\psi^{(M)}}{\partial w}(\mu_1 - (\mu_1+\lambda_1+\lambda_2)w + \lambda_1 w^2 + \lambda_2 wz) + \frac{\partial\psi^{(M)}}{\partial z}(\mu_2 - \mu_2 z),$$
$$\frac{\partial\psi^{(\not M)}}{\partial t} = \frac{\partial\psi^{(\not M)}}{\partial w}(\mu_1 - (\mu_1+\lambda_1+\lambda_2)w + \lambda_1 w^2 + \lambda_2 wz) + \frac{\partial\psi^{(\not M)}}{\partial z}(\mu_2 - \mu_2 z).$$

Initial condition: $\psi^{(M)}[w,z,0] = w, \quad \psi^{(\not M)}[w,z,0] = z.$

Note that as the forward equation is a linear PDE with only first order terms, the initial condition is sufficient for the uniqueness of solutions (see e.g. the chapter on Method of Characteristics, [6]). Given that initially $n_M(0) \sim \text{Bin}(n(0), p_M)$ (equivalently, $n(0) \sim \text{Poisson}(N)$ and $n_M(0) \sim \text{Poisson}(p_M \times N)$),

the p.g.f. of equation (10) becomes

$$\begin{aligned}\sum_j \binom{n(0)}{j} p_M^j (1-p_M)^{n(0)-j} g_{j,n(0)-j}[w,z,t] &= \sum_j \binom{n(0)}{j} (p_M s_M \ \ \psi^{(M)}[\frac{w}{s_M},\frac{z}{s_{\not M}},t])^j ((1-p_M) s_{\not M} \ \ \psi^{(\not M)}[\frac{w}{s_M},\frac{z}{s_{\not M}},t]) \\ &= \Big(p_M s_M \ \ \psi^{(M)}[\frac{w}{s_M},\frac{z}{s_{\not M}},t] + (1-p_M) s_{\not M} \ \ \psi^{(\not M)}[\frac{w}{s_M},\frac{z}{s_{\not M}},t]\Big)^{n(0)}.\end{aligned}$$

Note that whenever $g_{X,Y}[w,z] = \mathbb{E}[w^X z^Y]$ is the p.g.f. of the joint distribution of $X$ and $Y$, the p.g.f. of the sum $X+Y$ is $g_{X+Y}[z] = \mathbb{E}[z^{X+Y}] = \mathbb{E}[z^X z^Y] = g_{X,Y}[z,z]$. The p.g.f. of $n = n_M + n_{\not M}$ with $n_M(0) \sim \mathrm{Bin}(n(0), p_M)$ is therefore

$$g_{n(0)}[z,t] = \Big(p_M s_M \ \ \psi^{(M)}[\frac{z}{s_M},\frac{z}{s_{\not M}},t] + (1-p_M) s_{\not M} \ \ \psi^{(\not M)}[\frac{z}{s_M},\frac{z}{s_{\not M}},t]\Big)^{n(0)}. \qquad (15)$$

Now,

$$P\{T_G > t \,|\, n(0)\} = g_{n(0)}[1,t], \quad \text{and} \quad f_\tau[t+\tau] = \mathbb{E}[g_{n(0)}[1,t]]$$

where $g_{n(0)}[1,t]$ is given by (15), and $\psi^{(M)}$, $\psi^{(\not M)}$ are found numerically by solving the Kolmogorov equations.

## Expressions that relate the models' variables to RFI curves

Presented in this section are derivations of the expressions which relate the variable of the models, i.e. the number of micrometastases $n$ in a patient without growing or detectable metastases, to RFI curves and hazard rates.

## Models 1 and 3

In the following analysis we allow seeding and disappearance of micrometastases, as in Model 1, and a variable rate of the growth event, as in Model 3. Therefore, the following analysis applies to Model 1 in the special case $\kappa(t)$ is constant in time, and to Model 3 in the special case of no secondary metastasis ($\lambda = 0$) and no disappearance of micrometastases ($\mu = 0$). It is necessary to begin with a lemma.

*Lemma.*

Let $\mathbb{E}_{\not G}[(\cdot)(t)] = \mathbb{E}[(\cdot)(t)|T_G > t]$ be the expectation of variable $(\cdot)$ conditioned upon no growth event by time $t$. Then

$$\frac{d}{dt} P\{T_G > t\} = -\kappa(t)\ \mathbb{E}_{\not G}[n(t)]\ P\{T_G > t\},$$

and

$$\frac{d^2}{dt^2} P\{T_G > t\} = \mathbb{E}_{\not G}[\kappa(t)^2 n^2 - \kappa(t)\,(\lambda n_{_M} - \mu n) - \kappa'(t)\, n] P\{T_G > t\},$$

where the variable $n$ is the total number of micrometastases in a patient at time $t$, and $n_{_M}$ is the number of these in environments that permit secondary metastasis.

*Proof.*

$$\begin{aligned}
\frac{dP\{T_G > t\}}{dt} &= \lim_{\Delta t \to 0} \frac{P\{T_G > t+\Delta t\} - P\{T_G > t\}}{\Delta t} \\
&= \lim_{\Delta t \to 0} \frac{(P\{T_G > t+\Delta t | T_G > t\} - 1)P\{T_G > t\}}{\Delta t} \\
&= \lim_{\Delta t \to 0} \Big( \frac{\sum_u (1-\kappa(t)\, u\, \Delta t - o_u(\Delta t))P\{n(t) = u | T_G > t\} - 1}{\Delta t} \Big) \cdot P\{T_G > t\} \\
&= \lim_{\Delta t \to 0} \Big( \frac{1-\kappa(t)\,\Delta t\, \mathbb{E}_{\not{G}}[n(t)] - 1 - \sum o_u(\Delta t)}{\Delta t} \Big) P\{T_G > t\} = -\kappa(t)\, \mathbb{E}_{\not{G}}[n(t)]\ P\{T_G > t\}
\end{aligned}$$

as required, assuming in line three that the series $\sum_u o_u(\Delta t)$ converges uniformly in $\Delta t$ so that $\sum_u o_u(\Delta t) = o(\Delta t)$.

Here $n_{\not{M}} = n - n_M$ and, unlike in Model 3, $\kappa = \kappa(t)$ may depend on time. Let $P_{(j,k)\to(l,m)}(t)$ be the probability that $(n_M, n_{\not{M}})(t) = (l,m)$ given $(n_M, n_{\not{M}})(0) = (j,k)$. Let $\langle (\cdot)(t) \rangle$ denote expectation of $(\cdot)$ at time $t$ when the definition of $(n_M, n_{\not{M}})(t)$ is altered slightly: when $T_G \le t$, $(n_M, n_{\not{M}})(t) = (0,0)$, otherwise $(n_M, n_{\not{M}})(t)$ is as usual. Then

$$\begin{aligned}
\langle n_M(t+\Delta t) \rangle &= \sum_{l,m} \ \langle n_M(t+\Delta t) | (n_M, n_{\not{M}})(t) = (l,m) \rangle \ \ P_{(j,k)\to(l,m)}(t) + 0 \cdot P\{T_G \le t\} \\
&= \sum_{l,m} \Big( (l+1)p_M \lambda l \Delta t + l(1 - p_M \lambda l \Delta t - \mu l \Delta t - \kappa(l+m)\Delta t) + (l-1)\mu l \Delta t \\
&\qquad\qquad + 0 \cdot \kappa(l+m)\Delta t \Big) \times P_{(j,k)\to(l,m)}(t) + o_{l,m}(\Delta t) \\
&= \sum_{l,m} l P_{(j,k)\to(l,m)}(t) + p_M \lambda \sum_{l,m} l P_{(j,k)\to(l,m)}(t) \Delta t - \mu \sum_{l,m} l P_{(j,k)\to(l,m)}(t) \Delta t \\
&\qquad\qquad - \kappa \sum_{l,m} l(l+m) P_{(j,k)\to(l,m)}(t) \Delta t + o_{l,m}(\Delta t) \\
&= \langle n_M(t) \rangle + p_M \lambda \ \langle n_M(t) \rangle \ \Delta t - \mu \ \langle n_M(t) \rangle \ \Delta t - \kappa \ \langle n_M(t) n(t) \rangle \ \Delta t + \sum_{l,m} o_{l,m}(\Delta t)
\end{aligned}$$

where the second line is got by conditioning on the possible events in time $\Delta t$. Now the series $\sum_{l,m} o_{l,m}(\Delta t)$ is again assumed to converge uniformly in $\Delta t$ so that $\sum_{l,m} o_{l,m}(\Delta t) = o(\Delta t)$. Rearranging, and taking the limit $\Delta t \downarrow 0$, gives the ODE

$$\langle n_M \rangle' \ = \ p_M \lambda \ \langle n_M \rangle - \mu \ \langle n_M \rangle - \kappa \ \langle n_M n \rangle.$$

Similarly,

$$\langle n_{\not{M}}(t+\Delta t) \rangle \ = \ \langle n_{\not{M}}(t) \rangle + (1-p_M)\lambda \ \langle n_M(t) \rangle \ \Delta t - \mu \ \langle n_{\not{M}}(t) \rangle \ \Delta t - \kappa \ \langle n_{\not{M}}(t) n(t) \rangle \ \Delta t + o(\Delta t)$$

gives

$$\langle n_{\not{M}} \rangle' \ = \ (1-p_M)\lambda \ \langle n_M \rangle - \mu \ \langle n_{\not{M}} \rangle - \kappa \ \langle n_{\not{M}} n \rangle.$$

As $\langle n \rangle' = \langle n_M \rangle' + \langle n_{\not{M}} \rangle'$, from the ODEs above

$$\langle n \rangle' \ = \ \lambda \ \langle n_M \rangle - \mu \ \langle n \rangle - \kappa \ \langle n^2 \rangle.$$

For any $\alpha, \beta$ clearly

$$\left\langle n_{_M}^{\alpha} n^{\beta} \right\rangle = \left\langle n_{_M}^{\alpha} n^{\beta} | T_G > t \right\rangle P\{T_G > t\} + 0 \cdot P\{T_G \leq t\} \;=\; \mathbb{E}\Big[ n_{_M}^{\alpha} n^{\beta} | T_G > t \Big] P\{T_G > t\}$$
$$= \mathbb{E}_{\not G}\Big[ n_{_M}^{\alpha} n^{\beta} \Big] P\{T_G > t\}$$

for $P\{T_G > t\} > 0$. Therefore

$$\begin{aligned}
\frac{dP\{T_G > t\}}{dt} &= -\kappa \mathbb{E}_{\not G}[n(t)] P\{T_G > t\} = -\kappa \langle n \rangle \\
\Rightarrow \quad \frac{d^2 P\{T_G > t\}}{dt^2} &= -(\kappa' \langle n \rangle + \kappa \langle n \rangle'), \text{ and substituting the ODE for } \langle n \rangle', \\
\frac{d^2 P\{T_G > t\}}{dt^2} &= -\kappa' \langle n \rangle - \kappa(\lambda \, \langle n_M \rangle - \mu \, \langle n \rangle - \kappa \, \langle n^2 \rangle) \\
&= \Big( \kappa^2 \mathbb{E}_{\not G}[n^2] - \kappa(\lambda \mathbb{E}_{\not G}[n_{_M}] - \mu \mathbb{E}_{\not G}[n]) - \kappa' \mathbb{E}_{\not G}[n] \Big) P\{T_G > t\} \\
&= \mathbb{E}_{\not G}[\kappa^2 n^2 - \kappa(\lambda n_{_M} - \mu n) - \kappa' n] P\{T_G > t\}
\end{aligned}$$

where $\kappa'$ is the derivative of $\kappa$ with respect to time, defined for $t$ such that $P\{T_G > t\} > 0$. □

We are now ready to derive the expression presented in the Methods section of the main text.

Parameters may vary among patients: $f_\Psi$ denotes the p.d.f. of the set of parameters $\Psi$ among patients who do not have growing or detectable cancers upon resection. Then $f_\tau[t+\tau] = \int P\{T_G > t|\Psi\} f_\Psi d\Psi$, and

$$\begin{aligned}
h[t+\tau] &= -\frac{f_\tau'[t+\tau]}{f_\tau[t+\tau]} = -\frac{\int \frac{d}{dt} P\{T_G > t|\Psi\} f_\Psi d\Psi}{\int P\{T_G > t|\Psi\} f_\Psi d\Psi} = \int \mathbb{E}_{\not G}[\kappa(t)\, n(t)|\Psi] \cdot \frac{P\{T_G > t|\Psi\} f_\Psi d\Psi}{\int P\{T_G > t|\Psi\} f_\Psi d\Psi} \\
&= \mathbb{E}_t[\mathbb{E}_{\not G}[\kappa(t)\, n(t)|\Psi]] = \mathbb{E}_t[\kappa(t)\, n], \qquad (16)
\end{aligned}$$

by the Lemma, and where the expectation $\mathbb{E}_t[\cdot]$ and variance $\mathrm{Var}_t[\cdot]$ are over patients *without growing or detectable metastases at time t post-resection.* Similarly, $f_\tau''[t+\tau]/f_\tau[t+\tau] = \mathbb{E}_t[(\kappa(t)n)^2 - \kappa(t)(\lambda\, n_{_M} - \mu\, n) - \kappa'(t)n]$, which gets

$$\begin{aligned}
h'[t+\tau] &= -\Big( f_\tau''[t+\tau]/f_\tau[t+\tau] - (f_\tau'[t+\tau]/f_\tau[t+\tau])^2 \Big) \\
&= \mathbb{E}_t[\kappa\ (\lambda\ n_{_M} - \mu\ n) + \kappa' n] - \mathrm{Var}_t[\kappa\ n]. \qquad (17)
\end{aligned}$$

Note that these equations are independent of the initial condition.

**Model 2**

Again, it is necessary to begin with a lemma, the proof of which is very similar to the proof of the preceding lemma.

*Lemma.*

$$\begin{aligned}
\frac{d}{dt} P\{T_G > t\} &= -\mathbb{E}_{\not G}[\kappa_{SV}\, n_{SV} + \kappa_V\, n_V]\, P\{T_G > t\}, \qquad (18) \\
\frac{d^2}{dt^2} P\{T_G > t\} &= \mathbb{E}_{\not G}[(\kappa_{SV}\, n_{SV} + \kappa_V\, n_V)^2 - \kappa_S \kappa_{SV}\, n_S]\, P\{T_G > t\}. \qquad (19)
\end{aligned}$$

*Proof.*

$$
\begin{aligned}
\frac{d}{dt}P\{T_G > t\} &= \lim_{\Delta t\to 0}\frac{P\{T_G > t+\Delta t\} - P\{T_G > t\}}{\Delta t} \\
&= \lim_{\Delta t\to 0}\frac{(P\{T_G > t+\Delta t | T_G > t\} - 1)P\{T_G > t\}}{\Delta t} \\
&= \lim_{\Delta t\to 0}\Big(\frac{\sum_{u,v}(1-\kappa_{SV}\,u\,\Delta t - \kappa_V\,v\,\Delta t - o_{u,v}(\Delta t))P\{(n_{SV},n_V)(t)=(u,v)|T_G>t\} - 1}{\Delta t}\Big) \\
&\qquad\qquad \times \quad P\{T_G > t\} \\
&= \lim_{\Delta t\to 0}\Big(\frac{1-\kappa_{SV}\,\Delta t\,\mathbb{E}_{\mathcal{G}}[n_{SV}(t)] - \kappa_I\,\Delta t\,\mathbb{E}_{\mathcal{G}}[n_V(t)] - 1 - \sum o_{u,v}(\Delta t)}{\Delta t}\Big)P\{T_G > t\} \\
&= -(\kappa_{SV}\,\mathbb{E}_{\mathcal{G}}[n_{SV}] + \kappa_V\,\mathbb{E}_{\mathcal{G}}[n_V])\; P\{T_G > t\}
\end{aligned}
$$

as required, assuming in line three that the series $\sum_{u,v} o_{u,v}(\Delta t)$ converges uniformly in $\Delta t$ so that $\sum_{u,v} o_{u,v}(\Delta t) = o(\Delta t)$.

Let $P_{(a,b,c)\to(k,l,m)}(t)$ be the probability that $(n_S, n_{SV}, n_I)(t) = (k,l,m)$ given $(n_S, n_{SV}, n_V)(0) = (a,b,c)$. We use the same trick as before. Let $\langle(\cdot)(t)\rangle$ denote expectation of $(\cdot)$ at time $t$ when the definition of $(n_S, n_{SV}, n_V)$ is altered slightly: when $T_G \le t$, $(n_S, n_{SV}, n_V)(t) = (0,0,0)$, otherwise $(n_S, n_{SV}, n_V)(t)$ is as usual. Then equation (18) can be expressed as $\frac{d}{dt}P\{T_G > t\} = \kappa_{SV}\langle n_{SV}\rangle + \kappa_V\langle n_V\rangle$ and when $\kappa_{SV}$ and $\kappa_V$ are constant in time this gets

$$\frac{d^2}{dt^2}P\{T_G > t\} = -\kappa_{SV}\langle n_{SV}\rangle' - \kappa_V\langle n_V\rangle'$$

so to find $\frac{d^2}{dt^2}P\{T_G > t\}$ we need expressions for $\langle n_{SV}\rangle'$ and $\langle n_V\rangle'$. By the mathematical formulation of Model 2,

$$
\begin{aligned}
\langle n_{SV}(t+\Delta t)\rangle &= \sum_{(k,l,m)} \langle n_{SV}(t+\Delta t)\,|\,(n_S,n_{SV},n_V)(t)=(k,l,m)\rangle\cdot P_{(a,b,c)\to(k,l,m)}(t) + 0\cdot P\{T_G < t\} \\
&= \sum_{(k,l,m)} \Big((l+1)\kappa_S k\Delta t + l\,(1-\kappa_S\,k\Delta t - \kappa_{SV}l\Delta t - \kappa_V m\Delta t) \\
&\qquad + \quad 0\cdot(\kappa_{SV}l + \kappa_V m)\Delta t + o(\Delta t)\Big)\cdot P_{(a,b,c)\to(k,l,m)}(t) \\
&= \langle n_{SV}(t)\rangle + \sum_{(k,l,m)}((l+1)\kappa_S k - \kappa_S lk - \kappa_{SV}l^2 - \kappa_V lm)\Delta t\cdot P_{(a,b,c)\to(k,l,m)}(t) + o(\Delta t)
\end{aligned}
$$

which gives

$$\langle n_{SV}\rangle' = \kappa_S\langle n_S\rangle - \kappa_{SV}\langle n_{SV}^2\rangle - \kappa_V\langle n_{SV}\,n_V\rangle.$$

Similarly,

$$
\begin{aligned}
\langle n_V(t+\Delta t)\rangle &= \sum_{(k,l,m)} \langle n_V(t+\Delta t)\,|\,(n_S,n_{SV},n_V)(t)=(k,l,m)\rangle\cdot P_{(a,b,c)\to(k,l,m)}(t) + 0\cdot P\{T_G < t\} \\
&= \sum_{(k,l,m)} \Big(m\,(1-\kappa_{SV}l\Delta t - \kappa_V m\Delta t) + 0\cdot(\kappa_{SV}l + \kappa_V m)\Delta t + o(\Delta t)\Big)\cdot P_{(a,b,c)\to(k,l,m)}(t) \\
&= \langle n_V(t)\rangle + \sum_{(k,l,m)}(-\kappa_{SV}ml - \kappa_V m^2)\Delta t\cdot P_{(a,b,c)\to(k,l,m)}(t) + o(\Delta t)
\end{aligned}
$$

which gives

$$\langle n_V\rangle' = -\kappa_{SV}\langle n_{SV}\,n_V\rangle - \kappa_V\langle n_V^2\rangle.$$

Substituting the expressions for $\langle n_{SV}\rangle'$ and $\langle n_V\rangle'$ into the expression for $d^2P\{T_G > t\}/dt^2$ gets

$$\begin{aligned}\frac{d^2}{dt^2}P\{T_G > t\} &= -\kappa_S\,\kappa_{SV}\langle n_S\rangle + \langle(\kappa_{SV}n_{SV} + \kappa_V n_V)^2\rangle \\ &= \Big(-\kappa_S\,\kappa_{SV}\mathbb{E}_{\not{G}}[n_S] + \mathbb{E}_{\not{G}}[(\kappa_{SV}n_{SV} + \kappa_V n_V)^2]\Big)\,P\{T_G > t\},\end{aligned}$$

as required. □

The next step in the derivation is precisely analogous to the derivation following the lemma for Models 1 and 3 above. We have,

$$h[t+\tau] \,=\, \mathbb{E}_t[\kappa_{SV}n_{SV} + \kappa_V n_V], \quad h'[t+\tau] \,=\, \mathbb{E}_t[\kappa_S\,\kappa_{SV}\,n_S] \,-\, \mathrm{Var}_t[\kappa_{SV}\,n_{SV} \,+\, \kappa_V\,n_V]$$

where the expectation $\mathbb{E}_t[\cdot]$ and variance $\mathrm{Var}_t[\cdot]$ are over patients *without growing or detectable metastases at time t post-resection.* Note that again these equations are independent of the initial conditions.

## Supplementary Material for Results

### Unusual shapes including two late peaks in the hazard rate can be explained by the patient cohort being heterogeneous

Hazard rates can have slightly more exotic shapes other than being primarily flat or having one maximum followed by a continual decrease (see Figure 1; in this data set [7] there is also an earlier peak (not shown); this earlier peak can be accounted for by surgery stimulating the growth of metastases [8,9]). In Figure 1, we show that a heterogeneous patient cohort made up of mixed, unclassified cancer dormancy types, which can be represented by taking the weighted linear combination of distinct parameter sets in Models 1–3, can account approximately for such hazard rates. Parameters must satisfy the inequalities in results section *Maxima in hazard rates can be explained by an increase in the tumourigenicity of micrometastases* and be such that the hazard rates of different dormancy types peak at different times.

# References

1. Kusama S, Spratt JS, Donegan WL, Watson FR, Cunningham C (1972) The cross rates of growth of human mammary carcinoma. Cancer 30: 594–599.

2. Demicheli R, Terenziani M, Bonadonna G (1998) Estimate of tumor growth time for breast cancer local recurrences: rapid growth after wake-up? Breast Cancer Res Treat 51: 133–137.

3. Klein CA (2009) Parallel progression of primary tumours and metastases. Nat Rev Cancer 9: 302–312.

4. Willis L (2011) Three probabilistic models of phenomena in the life sciences. Ph.D. University College London, UK.

5. Karlin S, Tavaré S (1982) Linear birth and death processes with killing. J Appl Probab 19: 477–487.

6. Zwillinger D (1989) Handbook of Differential Equations. Academic Press.

7. Demicheli R (2001) Tumour dormancy: findings and hypotheses from clinical research on breast cancer. Semin Cancer Biol 11: 297–306.

8. Retsky MW, Demicheli R, Swartzendruber D, Bame P, Wardwell R, et al. (1997) Computer simulation of a breast cancer metastasis model. Breast Cancer Res Treat 45: 193–202.

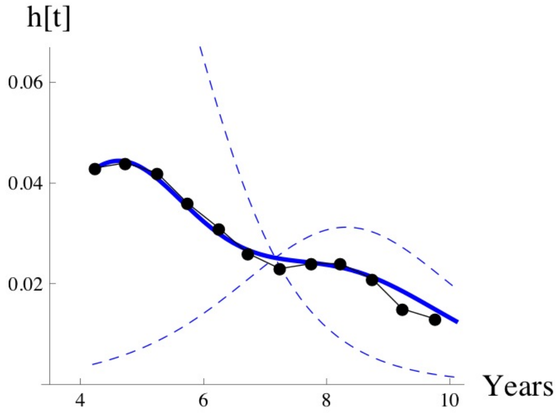


**Figure 1.** A hazard rate from post-resection breast cancer relapse-free data [7] (relapse-free data is black squares, left plot). Model 1 has been fitted to this two-peaked hazard rate (solid blue curve); a mixed cohort of two distinct but unclassified types of cancer was permitted, where each type corresponds a to distinct parameter set for Model 1: 42% of patients have parameters $\{\kappa, \lambda, \mu, p_M, N\} = \{0.11, 1.35, 0.38, 0.9, 0.5\}$, while 58% of patients have parameters $\{\kappa, \lambda, \mu, p_M, N\} = \{0.0062, 1.35, 0.41, 0.9, 0.25\}$. Dashed lines show the hazard rates for each type. For each type $(p_M\lambda - \mu)/\kappa > 1$ gives a peak; these combine to a hazard rate with two peaks. The parameters do not necessarily represent parameters of the cohort: other parameter values fit the data equally well.

9. Retsky M, Demicheli R, Hrushesky W, Baum M, Gukas I (2010) Surgery triggers outgrowth of latent distant disease in breast cancer: An inconvenient truth? Cancers 2: 305–337.
